# Supplementary material for: Content-rich biological network constructed by mining PubMed abstracts
Source: BMC Bioinformatics. 2004 Oct 8;5:147. doi: 10.1186/1471-2105-5-147 (PMC528731; doi:10.1186/1471-2105-5-147)
Supplement: Additional File 2 — The original results of the above study (non-essential files are deleted to keep the file size under the limit set by BMC bioinformatics). [file 1471-2105-5-147-S2.bz2 › chilibotAdditionalFile2/dip05/135ID7784054E480/html/TP53_RAF1.html]

 


 **TP53** and **RAF1** 
  
Found 9 abstracts in PubMed, retrieved 05.  
 

 What does Google say? 
 PDF only 
| .edu only 

---

**Interactive relationship** (e.g. stimulation, inhibition, etc)

**Non-interactive relationship** (e.g. studied together, co-existance, homology, etc.)

- However, in the ovarian cancer cell lines that have a mutant  **TP53** , the cytotoxicity induced by 60 nM paclitaxel exhibited the same relationship to Raf 1  [ **RAF1** ]  kinase activity as previously observed in cervical tumor cell lines.  Ref: 10854551 Oncol Rep< MedlineTA>Oncol Rep,
- In the two cell lines that possess a wild type  **TP53** , the relationship between Raf 1  [ **RAF1** ]  kinase activity and paclitaxel resistance was different from that observed in the cervical tumor cell lines.  Ref: 10854551 Oncol Rep< MedlineTA>Oncol Rep,
- Sustained activation of Ras Raf  [ **RAF1** ]  mitogen activated protein kinase cascade by the tumor suppressor p53  [ **TP53** ] .  Ref: 10890907 Proc Natl Acad Sci U S A, 2000
- Tumor suppressor p53  [ **TP53** ]  induction in response to cellular stresses activates the mitogen activated protein kinase MAPK cascade through pathways involving Ras and RAF  [ **RAF1** ]  p53 s ability to activate this pathway is dependent on p53 mediated transcription.  Ref: 11296226 EMBO J, 2001
